# Females’ scarcity of testosterone: a helper to save somatic resources in unfavourable environments. A Comment on: ‘The sexy and formidable male body: men’s height and weight are condition-dependent, sexually selected traits’ (2025), by Giofrè D *et al*.

**DOI:** 10.1098/rsbl.2025.0099

**Published:** 2025-06-11

**Authors:** Thomas Remer

**Affiliations:** ^1^ University of Bonn, Dortmund, Germany

**Keywords:** sexual size dimorphism, developmental health, human height, growth hormone, insulin-like growth factor-1 axis, oestrogens, testosterone

*Biol. Lett.* **21**: 20240565 (Published 22 January 2025). (https://doi.org/10.1098/rsbl.2024.0565)

## 1. Introduction

In their recent *Biology Letters* article, Giofrè *et al.* [1] examined changes in sexual size dimorphism (SSD) in humans. The authors used multinational survey data of sex differences in height and weight compiled by the World Health Organization (WHO) along with a measure, termed human development index assessing human well-being.

The main finding of their cross-sectional epidemiological study was that SSD is greater in more favourable environments. More precisely, increases in body height and weight going along with improved living conditions were markedly stronger in men than in women. Men’s gains in height and weight proved to be more than double those of women’s, demonstrating an increase in SSD.

The authors concluded that these findings—combining evolutionary biology with measures of human well-being—may provide novel insights into how socio-ecological factors and sexual selection shape key physical traits.

In a broader sense, this a reasonable interpretation if one recalls that sexual selection is defined as the evolutionary process that favours the increase in frequency of genes that confer a reproductive advantage [2] by increasing mating success [3]. What is clear is that the relevant genes involved regulate hormonal interactions with the growth hormone (GH) axis. However, it is not clear, and Giofrè *et al.* [1] did not comment on, what the underlying endocrine regulatory mechanisms are that cause differences in males’ and females’ changes in the SSD.

## 2. Role of oestrogens for the growth hormone/insulin-like growth factor-1 axis

In both males and females, oestrogens function as important regulators of growth, substantially impacting the secretion of GH and insulin-like growth factor-1 (IGF-1), of which the latter is one of the principal growth stimulators. Mean adult circulating GH concentrations and GH secretion levels are higher in women than in men [4,5]. This is, at least in part, due to higher oestrogen concentrations in females, which reduce GH action in the liver via inhibition of hepatic GH receptor signalling, thus reducing hepatic IGF-1 output and in turn increasing GH secretion via reduced feedback inhibition [6,7]. The markedly increasing GH concentration, tightly linked to the rising oestrogen production during advancing puberty in girls [7–9], is not primarily a reflection of a reduced IGF-1 feedback inhibition, but first and foremost indicates the stimulatory role of oestrogens on females’ pituitary GH release. This has among others been demonstrated through the initiation of the pubertal growth spurt in a 14-year-old female with aromatase deficiency after daily replacement with 20 µg ethinyl oestradiol [10,11]. Oestrogens in the pituitary and hypothalamus centrally aromatized from testosterone are also the direct stimulators of GH secretion in males. Inhibition of aromatase activity, i.e. reduction of the conversion of testosterone to oestradiol does accordingly reduce GH secretion and the corresponding IGF-1 production in normal men [12]. In humans, aromatase is highly expressed in the hypothalamus and the pituitary gland of females and males [7,13].

In brief, pre-clinical evidence strongly supports direct stimulation of GH secretion by oestrogens in the pituitary of both sexes [7]. Besides, oestrogens are responsible for the closure of epiphyseal growth plates in both sexes so that height growth comes to an end. However, growth stops several years earlier in girls than in boys due to girls’ higher oestrogen production in the course of puberty. Doubtless, this longer growth period of males is one of the reasons for the greater SSD in more favourable environments, as less frequent periods of unfavourable GH- and IGF-1-inhibiting events, i.e. longer periods without illnesses, infections and nutritional detriments can provide additional anabolic time periods with an improved GH and IGF-1 efficiency in males.

## 3. Additional role of testosterone

Testosterone, in contrast to oestrogens, raises GH and resultant IGF-1 levels not only centrally via oestrogen action but also increases anabolism via an additional stimulatory effect, i.e. stimulation of hepatic IGF-1 secretion [14,15]. The higher testosterone levels during puberty in boys can thus further efficiently contribute to an increased accretion of height and weight, if sickness- and malnutrition-related, testosterone-suppressing stressors present in a respective generation are absent in the following generation. Such strong stressors do of course reduce anabolic GH axis activity and sex hormones in both sexes. However, the stressor-dependent, specifically male-related reduction of testosterone’s additional stimulatory effect on hepatic IGF-1 production can generate additional height loss, or in the case of an improved environment, extra height gain in males compared with females. Furthermore, testosterone probably stimulates growth directly at the level of the epiphyseal chondrocytes [11,16].

Accordingly, strong environmental- and/or health-related stressors due to poor living conditions during growth can affect an additional (IGF-1-related) growth-relevant and stress-suppressible hormonal interaction that is specifically testosterone-dependent, explaining, at least in part, males’ higher vulnerability of height and weight changes to stressor-relevant events during growth.

Overall, this variation in SSD indicates that in the case of reduced resources and poor living conditions, a stronger reduction in males’ than in females’ body size may help to save resources for the affected population or species with less interference with the somatic condition in females.

With regard to nutritional influences on body size, it is worth mentioning that, apart from general malnutrition, insufficient protein intake, in particular, can adversely affect males’ and females’ adult height, with stronger consequences for males’ stature [17]. It has recently been shown in a biomarker-based, prospective, longitudinal study in healthy growing children and adolescents that a habitual high dietary protein intake, clearly exceeding dietary recommendations, is independently positively associated with adult height in females [18]. In a way, this could be seen as some compensation for females’ testosterone scarcity regarding somatic growth efficacy. However, total explained variation of adult height by early life factors, parental, pubertal and dietary data was 80% in females and only 68% in males in this latter study. The lack of data on males’ adolescent life-course testosterone-secretion status may have been, at least in part, responsible for this clearly lower overall explanation of men’s stature, very probably not allowing the discovery of some effect of high protein intake on men’s height.

## Data Availability

This article has no additional data.
